# G-protein-coupled receptor-associated A-kinase anchoring proteins AKAP5 and AKAP12: differential signaling to MAPK and GPCR recycling

**DOI:** 10.1186/1750-2187-3-19

**Published:** 2008-12-02

**Authors:** Jiangchuan Tao, Craig C Malbon

**Affiliations:** 1 Department of Pharmacology, School of Medicine, Health Sciences Center, State University of New York at Stony Brook, Stony Brook, NY 11794-8651, USA

## Abstract

**Background:**

A-kinase Anchoring Protein AKAP5 and AKAP12 both dock to the β_2_-adrenergic receptor, the former constitutively, the latter dynamically in response to activation of the receptor with agonist.

**Results:**

In the current work we analyze the ability of each AKAP to contribute to two downstream signaling events, the activation of mitogen-activate protein kinase and the resensitization/recycling of the internalized, desensitized β_2_-adrenergic receptor to the cell membrane. Although both AKAP share a large number of docking partners in common (*e.g*., β_2_-adrenergic receptor, protein kinases A and C, protein phosphatase-2B, and negatively-charged membrane phospholipids), AKAP5 and AKAP12 are shown to segregate with respect to activation of Erk1,2 and to resensitization/recycling of β_2_-adrenergic receptor. A431 cells were found to highly express AKAP12, but little of AKAP5. HEK293 cells, in contrast, were found to highly express AKAP5, but little of AKAP12. Suppression of the expression of AKAP5 in either A431 cells or HEK293 cells leads to loss of the ability of the β_2_-adrenergic receptor to activate Erk1,2. Suppression of the expression of AKAP12 in either cell line leads to loss of the ability of these cells to resensitize the β_2_-adrenergic receptor.

**Conclusion:**

Knock-down experiments of endogenous AKAP 5 and AKAP12 in two cell lines used commonly to study β_2_-adrenergic receptor signaling clearly discriminate between the activation of mitogen-activated protein kinase (a downstream read-out solely mediated by AKAP5) and receptor recycling (a downstream read-out solely mediated by AKAP12).

## Background

The identification of a class of proteins harboring a binding site for the regulatory subunits (*i.e*., RI/RII) of cyclic AMP-dependent protein kinase A (PKA, A-kinase) was seminal in our understanding of the roles of these scaffold proteins, termed A-Kinase Anchoring Proteins or AKAPs, in cellular signaling [1]. The ability of AKAPs to dock PKA was followed by the discovery that AKAPs can act as molecular "tool boxes" that are multivalent and capable of docking PKA, protein kinase C (PKC), as well as phosphoprotein phosphatases, such as protein phosphatase-2B [2]. AKAPs have been shown to participate in macromolecular signaling complexes that include protein kinases (serine/threonine and tyrosine kinases), phosphatases, phosphodiesterases (PDE), adaptor molecules, ion channels, and also at least one member of the superfamily of G protein-coupled receptors (GPCR) [3]. Two AKAPs that associate with the prototypic GPCR, the β_2_-adrenergic receptor, have been the focus of intense research. Herein, we examine these two members of the class of GPCR-associated AKAPs, namely AKAP5 (also known as AKAP79/150) and AKAP12 (also known as gravin and AKAP250), comparing and contrasting structure/function, conserved domains and motifs, and details about their roles in two well known cellular signaling responses. We elucidate the role of each of these AKAP "molecular tool boxes" in mediating mitogen-activated protein kinase activation and in mediating GPCR resensitization and cyclic AMP generation.

## Results

**Figure 1:**
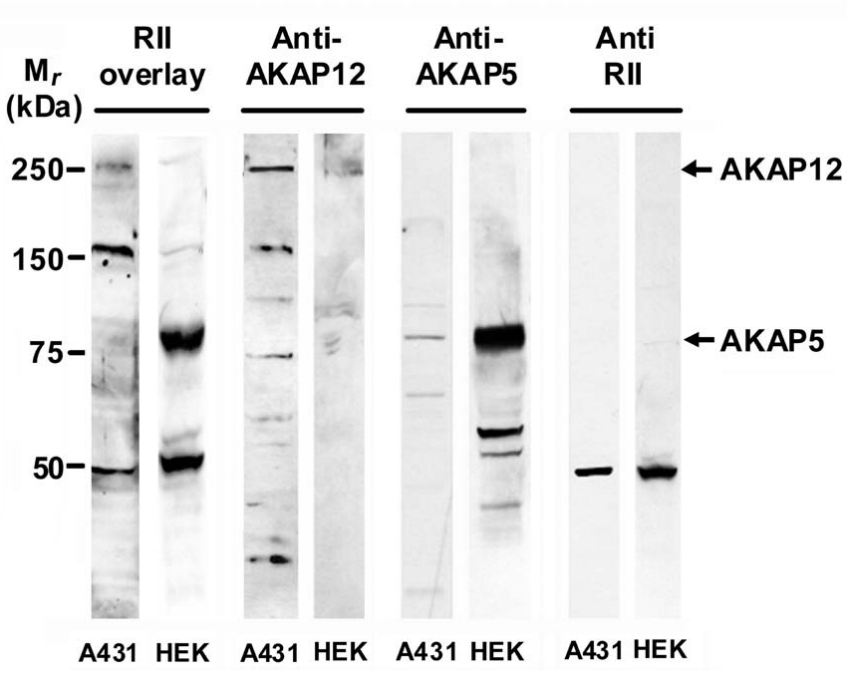
**Overlay analysis of A kinase-anchoring proteins in A431 and HEK cells**. Cell lysates (50 μg of protein/lane) of A431 or HEK cells were subjected to SDS-polyacrylamide gel electrophoresis, the resolved proteins transferred to nitrocellulose blots, and the blots subjected to renaturation and stained with A-kinase RII subunit for 2 h at room temperature. After washing, the presence of AKAPs was detected using an goat anti-RII antibody. The resultant blots also were stained with antibodies specific for AKAP12 or AKAP5.

AKAP5 and AKAP12 are molecular tool boxes that dock to GPCR, *e.g*., β_2_-adrenergic receptor. In view of their many common properties, including the docking to GPCR, we probed if AKAP5 and AKAP12 shared common functions in downstream signaling. We made use of two cell lines often employed in studies of one or the other AKAP [4-10], the human embryonic stem cell (HEK293) and the human epidermoid carcinoma cell (A431). We sought to evaluate the relative levels of expression of both AKAP5 and AKAP12 in these two well-known cell lines employed in studies of cell signaling, particularly signaling via GPCRs. The most informative identification of and assay of abundance of AKAPs is the use of the A-kinase overlay assay (fig. 1). In this assay, equivalent amounts of cellular protein are subjected to SDS-PAGE, the resolved proteins transferred to blots, and the presence of AKAPs identified by overlaying the blots with A-kinase RII α-subunit [11]. For the A431 cells, AKAP12 (250 kDa-*M*_*r*_) plus a ~170 kDa-*M*_*r *_proteolytic AKAP12 fragment were dominant species in the overlay assay and readily detected also in companion immunoblotting of the same samples using anti-AKAP12 antibodies. What is equally obvious is that for HEK293 cells, AKAP5 (79 kDa-*M*_*r*_) is readily stained by the overlay assay, an observation confirmed in the immunoblotting of the same samples using anti-AKAP5 antibodies (fig. 1). Perhaps even more of interest is not only that these cells lines express a dominant AKAP, but in the case of the A431 cells, AKAP12 is dominant, whereas AKAP5 is expressed to a relatively minor level. For HEK293 cells, the expression levels of these two AKAPs clearly were reversed, *i.e*., AKAP5 is the dominant AKAP and AKAP12 is expressed at markedly lower relative levels. Thus we can approach analysis of AKAP function for these two AKAPs, making use of cell lines that natively are replete in one and largely deficient of the other AKAP (fig. 1).

**Figure 2:**
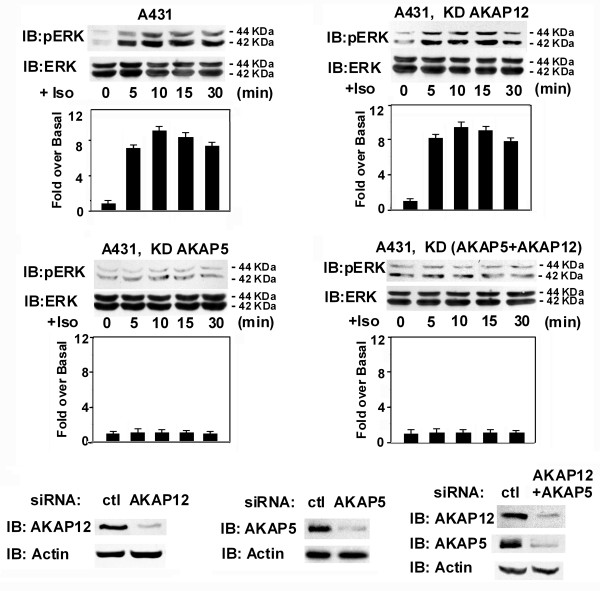
**Activation of the mitogen-activated protein kinase cascade to Erk1,2 in human epidermoid A431 cells in response to isoproterenol stimulation**. Wild-type 431 or A431 cells treated with siRNAs targeting either AKAP12 or AKAP5, or both AKAPs, were stimulated by isoproterenol (10 μM) for different intervals. Cell lysates were subjected to SDS-polyacrylamide gel electrophoresis, the resolved proteins transferred to nitrocellulose. The blots were stained by antibody against activation-specific phospho-Erk1,2, against pan-Erk, or against AKAP12 and AKAP5.

One of most interesting signaling outputs for β_2_-adrenergic receptors is the initiation of the mitogen-activated protein kinase cascade that leads to activation of Erk1,2. We assayed the activation of Erk1,2 in whole-cell homogenates by use of immunoblotting with activation-specific, phospho-specific antibodies to Erk1,2 (fig. 2). Treating wild-type A431 cells with the β_2_-adrenergic agonist isoproterenol (10 μM) for 0–30 min provokes a clear and marked activation of Erk1 (pErk1 = 44 kDa-*M*_*r*_) and Erk2 (pErk2 = 42 kDa-*M*_*r*_), while not changing the relative levels of either Erk1 or Erk2. Within 5 min of challenge with isoproterenol, levels of activated pERK1,2 increase markedly, a response that peaks at 10 min and thereafter slowly declines.

Since wild-type A431 cells express both AKAP5 (minor) and AKAP12 (dominant), we sought to determine in these cells which of the AKAPs is involved in mediating the signaling from the β_2_-adrenergic receptor to Erk1,2 activation. To address this question we made use of siRNA designed to suppress (*i.e*., knock-down, KD) the expression of either hAKAP5 or hAKAP12. As observed in the immunoblots of whole-cell homogenates stained with anti-AKAP12 antibodies, siRNA treatment knocked-down the expression of AKAP12 by more than 95%. The ability of isoproterenol to stimulate activation of Erk1,2 was largely unaffected by the nearly total suppression of the dominant AKAP12 in this cell line. siRNAs designed to knock-down AKAP5 were equally effective in suppressing expression of AKAP5, as shown in an overexposure of an immunoblot of AKAP5 expression in whole-cell homogenates of A431 cells. The ability of isoproterenol treatment to activate Erk1,2 in A431 cells was abolished when the expression of AKAP5, but not AKAP12, was knocked down. The effects of simultaneous knock-down of AKAP5 and AKAP12 displayed the same ability to block Erk1,2 activation as did knock-down of AKAP5 alone.

**Figure 3:**
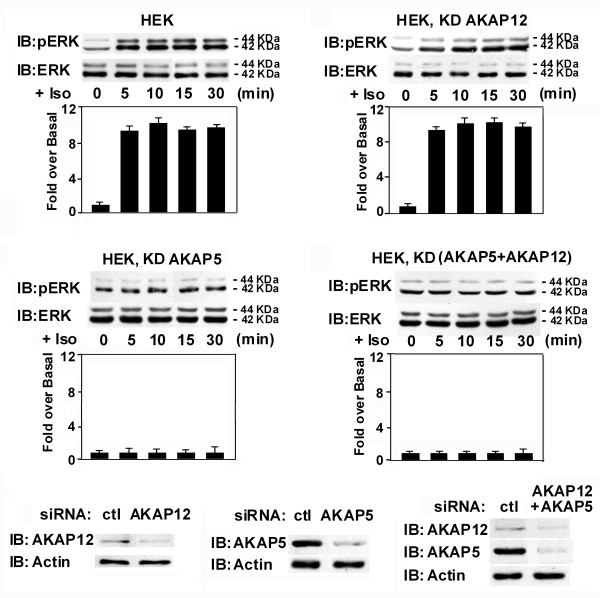
**Activation of the mitogen-activated protein kinase cascade to Erk1,2 in human embryonic kidney 293 cells in response to isoproterenol stimulation**. Wild-type HEK293 cells or HEK293 cells treated with siRNAs targeting either AKAP12 or AKAP5, or both AKAPs, were stimulated by isoproterenol (10 μM) for different intervals. Cell lysates were subjected to SDS-polyacrylamide gel electrophoresis, the resolved proteins transferred to nitrocellulose. The blots were stained by antibody against activation-specific phospho-Erk1,2, against pan-Erk, or against AKAP12 and AKAP5.

In HEK293 cells, identical experiments were performed. The main difference being that the HEK cells have just the opposite relative distribution of AKAP5 (dominant) and of AKAP12 (minor). HEK293 cells displayed a rapid (≤ 5 min) and robust Erk1,2 activation in response to isoproterenol (fig. 3). siRNA-induced knock-down effectively suppressed the expression of one or both of the AKAPs in these cells. Again, as in the A431 cells, the knock-down of AKAP12 in HEK293 cells had no effect on the ability of isoproterenol to activate Erk1,2. Suppression of AKAP5, in contrast, again blocks the Erk1,2 activation in response to isoproterenol, both in cells which express either high (HEK293 cells) or low (A431 cells) levels of endogenous AKAP5 (fig. 3). Thus in both A431 and HEK293 cells, AKAP5, but not AKAP12, mediates activation of the mitogen-activated protein kinase cascade in response to β-adrenergic stimulation.

**Figure 4:**
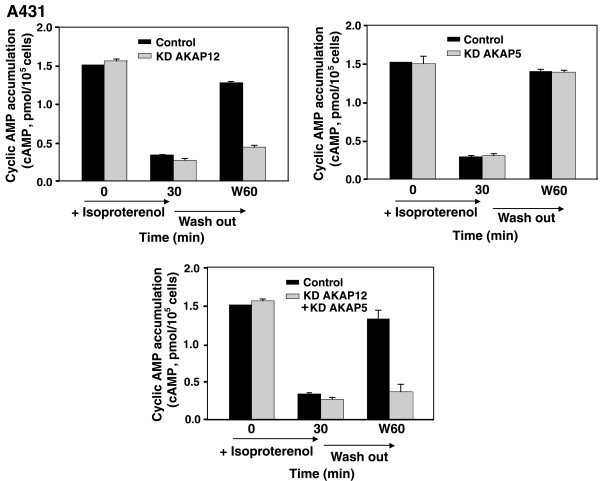
**Desensitization and Resensitization of β_2_AR in wild-type A431 cells and cells treated to knock-down AKAP12 or AKAP5**. *A*, cyclic AMP accumulation of β_2_AR-mediated cyclic AMP accumulation in A431 cells that were desensitized by 30 min treatment with Iso and then washed free of agonist for 60 min (*W60*) to permit resensitization was examined. Agonist-stimulated (*Iso*,10 μM) cyclic AMP accumulation was measured in untreated cells, in cells following a 30-min prior stimulation with Iso, and in cells treated with Iso for 30 min, washed free of agonist, incubated for 60 min and then assayed (*W60*). A431 cells were pretreated either with a control, scrambled sequence siRNA (Control) or with siRNA targeting AKAP12/AKAP5 to knock down (*KD*) the expression of endogenous AKAP12 or AKAP5. Cells were challenged with a β_2_-adrenergic agonist (*Iso*, 10 μM) for 30 min to provoke agonist-induced desensitization and internalization of β_2_AR. The recovery from agonist-induced desensitization, termed resensitization, was measured in cells after a 30-min challenge with Iso, and in Iso-treated cells that were washed free of agonist for 60 min (*W60*). Isoproterenol-stimulated cyclic AMP accumulation was measured in these cells and is reported as picomol of cyclic AMP accumulated per 10^5 ^cells. The results, displayed as mean ± S.E., are derived from at least three separate experiments performed with as many separate cultures of A431 cells. *, *p *≤ 0.01 for the difference from the amount of cyclic AMP accumulation measured in the control cells at 60 min following a washout of the agonist (*W60*).

Another function first observed to be mediated by AKAP12 (gravin) is the resensitization and recycling of the β_2_-adrenergic receptor. AKAP12 does not function in either the agonist-specific desensitization or the internalization of GPCRs [3,12,13]. Rather, once desensitization and internalization of β_2_-adrenergic receptors is complete, AKAP12 plays an essential function in the resensitization (dephosphorylation) of the receptors and in the recycling of the receptors from intracellular vesicles, mobilizing them to the cell membrane [12]. With respect to stimulation of cyclic AMP accumulation (over a 5 min period), wild-type A431 cells treated with isoproterenol for 30 min are fully "desensitized" to a second stimulation with the β-adrenergic agonist (+ Isoproterenol, 30 min, fig. 4). If the agonist is washed away, the cells nearly fully recover within the next 30 min (Wash out, W60 min, fig. 4). Thus, the cells have a robust stimulation of cyclic AMP, display agonist-induced desensitization, and recovery to the control response within 30 min of a wash-out of the agonist (fig. 4).

When A431 cells, replete in AKAP12 but not AKAP5, were treated with siRNA to knock-down the expression of AKAP12 (KD AKAP12), the ability of the cells to recover their cyclic AMP response to isoproterenol following a washout of agonist was largely blunted (fig. 4). Similar treatment of the cells with siRNA targeting expression of AKAP5 (KD AKAP5), in contrast, had no effect on the ability of the cells to recover their cyclic AMP response (fig. 4). When cells were simultaneously treated with siRNAs targeting both AKAP5 and AKAP12, the recovery of the cells from agonist-induced desensitization following a wash-out was nearly abolished, much like the response of the cells to siRNA targeting the AKAP12 only (fig. 4). These data clearly demonstrate that expression of AKAP12, but not AKAP5, is essential to the ability of cells to recover from agonist-induced desensitization. AKAP 5 appears to play no significant role in this AKAP function.

**Figure 5:**
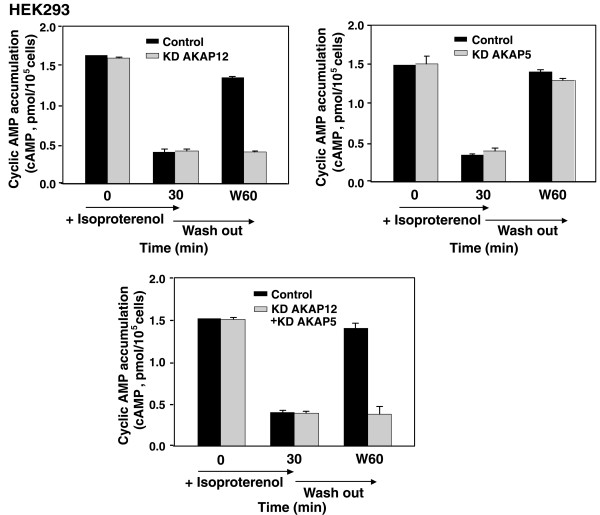
**Desensitization and Resensitization of β_2_AR in wild-type HEK293 cells and cells in which AKAP12 or AKAP5 were knocked-down**. *A*, cyclic β_2_AR-mediated cyclic AMP accumulation in HEK cells that were desensitized by 30 min treatment with Iso and then washed free of agonist for 60 min (*W60*) to permit resensitization was examined. Agonist-stimulated (*Iso*,10 μM) cyclic AMP accumulation was measured in untreated cells, in cells following a 30-min prior stimulation with Iso, and in cells treated with Iso for 30 min, washed free of agonist, incubated for 60 min and then assayed (*W60*). HEK cells were pretreated with either a control, scrambled sequence siRNA (Control) or with siRNA to AKAP12 or AKAP5 to knock down (*KD*) the expression of endogenous AKAP12 or AKAP5. Cells were challenged with a β_2_-adrenergic agonist (*Iso*,10 μM) for 30 min to provoke agonist-induced desensitization and internalization of β_2_AR. The recovery from agonist-induced desensitization, termed resensitization, was measured in cells after a 30-min challenge with Iso, and in Iso-treated cells that were washed free of agonist for 60 min (*W60*). Isoproterenol-stimulated cyclic AMP accumulation was measured in these cells and is reported as picomol of cyclic AMP accumulated per 10^5 ^cells. The results, displayed as mean ± S.E., are derived from at least three separate experiments performed with as many separate cultures of A431 cells. *, *p *≤ 0.01 for the difference from the amount of cyclic AMP accumulation measured in the control cells at 60 min following a washout of the agonist (*W60*).

Parallel studies were performed in the HEK293 cells in which AKAP5 expression is highly abundant and AKAP12 is considerably less (figs. 1, 5). The cyclic AMP response of the HEK293 cells to initial stimulation, but no pre-incubation with isoproterenol (fig. 5) was similar to that observed in the A431 cells (fig. 4). Likewise, pretreatment with the β-adrenergic agonist provoked a marked desensitization to a subsequent challenge with isoproterenol. The agonist-induced desensitized state of the HEK293 cells was largely reversed once the cells were washed free of agonist and allowed to recover for 30 min. The siRNA-induced knock-down of AKAP12 in HEK293 cells had no effect on the initial stimulatory cyclic AMP response to agonist and did not influence the extent to which the cells were desensitized by prior treatment with agonist for 30 min (fig. 5). Suppression of AKAP12 (KD AKAP12) clearly abolished the ability of these cells to recover from agonist-induced desensitization, once the agonist was washed free (fig. 5). Parallel experiments performed using siRNAs targeting AKAP5 expression (KD AKAP5) had no effect on the stimulatory response, the ability of the cells to display agonist-induced desensitization, as well as recovery and resensitization from agonist-induced desensitization once the HEK293 cells were washed free of agonist (fig. 5). Treatment with siRNAs individually targeting both AKAP5 and AKAP12 provoked a loss in the ability of these cells to recover and to re-sensitize from agonist-induced desensitization much like that observed when on the AKAP12 was knocked-down. Thus, even in the cells which natively express relative high levels of AKAP5 and low levels of AKAP12 (fig. 1), AKAP12 appears to be playing the dominant role of catalyzing the recovery of the cells from agonist-induced resensitization. In the absence of AKAP12, normal recovery and resensitization does not occur following wash-out of the β-adrenergic agonist.

## Discussion

The functional roles of AKAPs in general [14] and the GPCR-associated AKAP specifically [3] are just beginning to be described in fine detail. AKAP5 and AKAP12 are early members of the AKAP family, a family that continues to expand its membership. AKAP5 and AKAP12 share an impressive list of properties in common: dock PKA RII subunit (by definition), protein phosphatase-2B, and protein kinase C; display three N-terminal, small positively-charged domains (PCD) that electrostatically bind to the inner leaflet of the lipid bilayer; and, dock to a prominent member (and likely others) of the superfamily of GPCR, the β_2_-adrenergic receptor [15]. In the current study we focus on two centrally important functions in which AKAPs have exerted functions in organizing downstream signaling: activation of the mitogen-activated protein kinase cascade terminating in Erk1,2 activation; and, catalysis of the resensitization and recycling of the GPCRs that have been subjected to agonist-induced desensitization. Since AKAP5 and AKAP12 have been implied to have similar roles and each has shown the capacity to interact with the β-adrenergic receptor we compared these two AKAPs for there ability to catalyze one or the other, or both, of these functional read-outs.

The studies were performed in two cell types that are commonly used to study cell signaling, especially GPCR-mediated activation of cyclic AMP accumulation and of the MAP kinase cascade. Of interest were the results of the initial characterization of these two cell lines with respect to the expression of the two AKAPs targeted in these studies. It would be challenging to compare the expression of these two different scaffolds if they did not share a common docking partner that appears to bind in a molar 1:1 manner and stoichiometrically. Using A-kinase RII subunit to probe blots from similar amount of whole-cell homogenates of HEK293 and A431 cells, we were able to demonstrate a remarkable difference between these cell lines, long favored in cell signaling studies of the cyclic AMP response [10,16]. HEK293 and A431 cells differ markedly with respect to expression of AKAP5; by A-kinase RII staining in the former, AKAP5 (79 kDA-*M*_*r*_) is shown to be the dominant AKAP family member expressed, whereas in the latter AKAP5 is shown to be only a minor AKAP. With respect to AKAP12, its abundance (including the 250 kDa-*M*_*r *_parent as well as the ~170 kDa-*M*_*r *_proteolytic fragment) is high in A431 cells and low in HEK293 cells. Thus in choosing to analyze the function of AKAP5 and AKAP12 in these two cell lines, we are probing conditions in which natively one AKAP form is dominant, while the other is a relatively minor species. In both cases, siRNA-induced knock-down of targeted AKAP was effective, regardless of whether it was the major or minor AKAP in the cell line under study.

**Figure 6:**
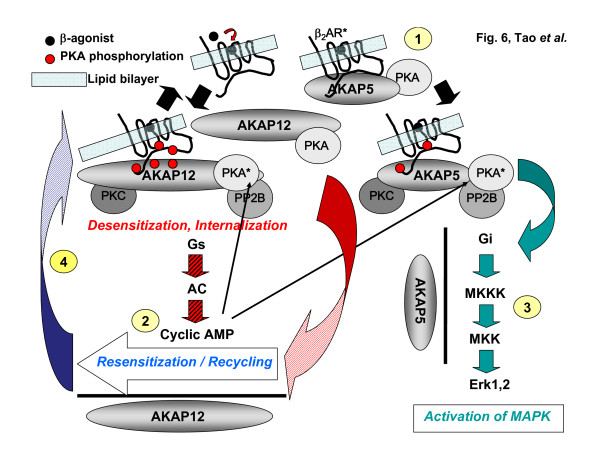
**Schematic of GPCR-associated AKAP79 and AKAP250: regulation of downstream signaling by beta_2_-adrenergic receptors**. The AKAPs provide multivalent docking sites for PKA, PKC, and PP2B as well as other protein kinases, phosphoprotein phosphatases, and adaptor molecules (not shown). Binding of the beta-adrenergic agonist leads to activation of the receptor (β_2_AR*, #1) and activation of Gs > adenylylcyclase activation > cyclic AMP accumulation > PKA activation (#2). The association of AKAP250 with the receptor is markedly enhanced by PKA phosphorylation of the receptor and scaffold, whereas AKAP79 association with the receptor is constitutive. AKAP79 mediates downstream signal switching from Gs to Gi-mediated activation of the mitogen-activated protein kinase cascade leading to Erk1,2 (#3). AKAP250 mediates the resensitization and recycling of the desensitized and internalized receptor (#4). Through docking of critical enzymes, the AKAP provide high spatial resolution and localization of downstream signaling. See the text for more details.

The outcome was remarkable in one major sense, the functional properties of AKAP 5 and AKAP12 with regard to the two prominent read-outs was very clear. AKAP5 was essential for expression of the ability of the GPCR β-adrenergic receptor to mediate the activation of the MAP kinase cascade to the level of Erk1,2, whereas AKAP12 was truly dispensable. This result was confirmed by studies using both cell lines. AKAP12, in contrast, was found to be essential for the ability of the cells to recover from agonist-induced desensitization (a hallmark for virtually all members of the GPCR superfamily of receptors) and yield re-sensitized, recycled receptors to the cell membrane. Again, this result was confirmed by studies using both cell lines (fig. 6). Thus, in spite of the many properties that AKAP5 and AKAP12 share, with respect to mediating the activation of the mitogen-activated protein kinase cascade to the level of Erk1,2 as well as with respect to mediating the recovery, resensitization and recycling of agonist-induced desensitized receptors these two AKAPs display *no *redundancy in function. Both AKAPs display docking of many partners in common (*e.g*., A-kinase RII subunit, protein kinase C, and the β-adrenergic receptor), then it must be the temporal sequence of docking, the dynamics of docking, or the docking of some partners not shared in common responsible for this difference in function. The most obvious insight to the basis for this lack of redundancy in these two downstream read-outs is the report that AKAP12 docks to the GPCR in a dynamic manner [2,3,13], whereas AKAP5 has been reported to dock constitutively [4]. The receptor-binding domain (RBD) of AKAP12 has been characterized structurally and is known to possess multiple A-kinase sites of phosphorylation [13]. AKAP5 has a sequence with some analogy to the AKAP12 RBD, which also includes sites (potential) for A-kinase phosphorylation [15], but this putative AKAP5 RBD needs careful and thorough characterization before we can rule the differences in the RBD to be important, or not, in the unique downstream signaling mediated by AKAP12 versus AKAP5.

The other avenue worthy of careful investigation is the trafficking of the AKAPs and the β-adrenergic receptors during receptor activation, desensitization, internalization, recovery, resensitization, and recycling to the cell membrane. Differences in the trafficking patterns of the AKAP may reveal how these two central functions of AKAP5 and AKAP12 can be compartmentalized in cells replete with both as well as deficient in one. Although the domain of the β_2_-adrenergic receptor that is the site of docking of AKAP12 (through the RBD) has been determined, one cannot assume that both AKAP12 and AKAP5 share the same docking site on the GPCR. If the dock site of the GPCR for these two AKAPs was common, one might expect that there would be some differences perhaps in the properties of the responses between cells in which one AKAP is the dominant form, while the other AKAP is a minor form. If the binding site on the GPCR were common and capable of interacting with either AKAP, overexpression of one AKAP (even natively) might be expected to act as a dominant negative of the function of the other AKAP. Knock-down studies of AKAP5/12 function in which we suppressed the expression of a single AKAP were compared to parallel studies in which we simultaneously suppressed the expression of both scaffolds. The results of this comparison do not support the notion that AKAP5 and AKAP12 share the same site of interaction on the GPCR. Clearly the roles of each AKAP as well as their possible interaction must be considered and probed to further delineate the functional roles of these interesting scaffold molecules.

## Conclusion

Human epidermoid carcinoma A431 cells and human embryonic kidney 293 cells express a different ratio of two AKAPs that are known to dock to G protein-coupled receptors. A431 cells natively express a variety of AKAP family members, AKAP12 being dominant in expression and AKAP5 being minor. HEK293 cells, in contrast, natively express relatively high amounts of AKAP5 and comparatively little AKAP5. We examined two well known read-outs (*i.e*., activation of Erk1,2 and the resensitization/recycling of G protein-coupled receptors) that are downstream to and regulated by the β_2_-adrenergic receptor, a prototypic G protein-coupled receptor. The beta-adrenergic agonist isoproterenol stimulates these two downstream responses in both cell types. Knock-down of AKAP5 abolishes the Erk1,2 activation; knock-down of AKAP12 blocks the ability of the cells to resensitize and recycle the internalized/desensitized β_2_-adrenergic receptor back to the cell membrane. This study reveals an unexpected discrimination by AKAP5/AKAP12 among downstream signaling pathways from G protein-coupled receptors. Both AKAP scaffolds share many docking partners (*e.g*., protein kinases A and C, phosphoprotein phosphatase-2B, β_2_-adrenergic receptor, and adaptor molecules) as well as interact dynamically with the cell membrane, yet cleanly discriminate between Erk 1,2 activation and receptor resensitization/recycling.

## Methods

### Cell culture

Human epidermoid carcinoma cells (A431) and human embryonic kidney cells (HEK293) were maintained in Dulbecco's modified Eagle's medium supplemented with 10% fetal bovine serum (HyClone, Logan, UT), penicillin (60 μg/ml), and streptomycin (100 μg/ml) and grown in a humidified atmosphere of 5% CO2 and 95% air at 37°C.

### siRNA-induced knock-down of AKAP5 and AKAP12

siRNAs targeting human AKAP5 (aagagaucagcagaagguagu) corresponding to nucleotides 48–68 or a "scrambled" siRNA control (aaggcaacaaaggcuaaguca) as well as those targeting human AKAP12 (cgaggcggcgccagacaccac) corresponding to nucleotides 161–181 or a "scrambled" siRNA control (ggagcgcggcgaccaacccca) were synthesized by Invitrogen (Calsbad, CA). siRNAs were transfected at a concentration of 50–100 nM into A431 or HEK-293 by the Lipofectamine 2000™ transfection reagent (Invitrogen). After 2 days, cell extracts were probed to determine the expression of each AKAP, so as to assess both the extent of the siRNA-induced suppression and specificity.

### Analysis of expression of AKAPs

Following siRNA-induced suppression of AKAP expression, the amount of residual AKAP was probed by immunoblotting. Cells were harvested and lysed in a lysis buffer (1% Triton X-100, 0.5% Nonidet-40, 10 mM dithiothreitol, 5 μg/ml aprotinin, 5 μg/ml leupeptin, 100 μg/ml bacitracin, 100 μg/ml benzamidine, 2 mM sodium orthovanadate, 150 mM NaCl, 5 mM EDTA, 50 mM NaF, 40 mM sodium pyrophosphate, 50 mM KH_2_PO_4_, 10 mM sodium molybdate, and 20 mM Tris-HCl, pH 7.4) at 4°C for 20 min. The samples were subjected to SDS-PAGE and the resolved proteins transferred electrophoretically to nitrocellulose blots. The blots were stained with primary antibodies (anti-AKAP79 from Upstate; anti-gravin kindly provided by Dr. John Scott) and detected with a secondary antibody. Immune complexes were made visible with ECL staining [17].

### Immunoblotting and quantification analysis of proteins

Proteins (60–100 μg samples) were analyzed by SDS-polyacrylamide gel electrophoresis and transferred electrophoretically to an Immobilon membrane (Millipore, Bedford, MA). The blots were stained with the primary antibodies indicated. Immunoreactive bands were detected using a horseradish peroxidase-conjugated, secondary antibody in tandem with ECL chemiluminescence. Exposed films were scanned by calibrated Umax 1000 absorbance scanner equipped with SilverFastAi software (LaserSoft Imaging Inc. Longboat Key, FL). The bands were quantified by use of *Aida*^® ^software (Raytest, Germany) [18].

### Mitogen-activated protein kinase assay

To assay Erk1,2, whole-cell homogenates were subjected to SDS-PAGE, as described above, and the blots then were probed with specific antibodies that cross-react with phosphorylated-activated forms of Erk1,2 (Thr202/Tyr204) or pan-ERK, to establish Erk protein levels. Use of secondary antibodies, staining, detection, and the quantification were as described above.

### RII overlay assay

Fifty micrograms of cell lysate or membrane protein was separated by electrophoresis on an SDS-polyacrylamide gel and transferred electrophoretically to nitrocellulose blots. The blots first were blocked with a Tris-buffered saline solution containing 10% heat-inactivated horse serum and then incubated with RII subunit for 2 h at room temperature. After washing, the presence of AKAPs was detected using an goat anti-RII α antibody, as described in immunoblotting analysis [3].

### Protocols for desensitization and resensitization of β_2_AR

Two days prior to the analysis of agonist-induced desensitization, the A431 cells were seeded in 96-well microtiter plates at a density of 25,000–50,000 cells/well. Routinely cells were serum-starved overnight, prior to analysis. For the current studies the cells were sampled for their capacity to accumulate cyclic AMP over a 5-min interval in response to 10 μM isoproterenol in one of three standard protocols [19]: cells with no extended pre-incubation with isoproterenol (+ Isoproterenol, "0" time); cells with 30 min of prior incubation with isoproterenol, briefly washed and then stimulated with the standard 5 min period of cyclic AMP accumulation (+ Isoproterenol, "30" time); and, cells pre-treated with isoproterenol for 30 min, washed-free of agonist, and allowed to resensitize for an additional 30 min (+ Isoproterenol > wash out = W60). The first protocol is the "control" for β-adrenergic stimulated accumulation of cyclic AMP, the second protocol is assay for β-adrenergic stimulated accumulation of cyclic AMP in the agonist-induced "desensitized" state, the third protocol is the assay for β-adrenergic stimulated accumulation of cyclic AMP in the recovered, "resensitized" state. Prior challenge with a β-adrenergic agonist severely blunts cyclic AMP accumulation in response to a second challenge and this loss in the cyclic AMP response is accompanied by a decline in the number of cell-surface receptors, as the desensitized receptors are sequestered and internalized. Thirty minutes following wash-out of the agonist, these desensitized receptors are fully resensitized and are recycled to the cell membrane. Likewise the cells subjected to wash-out and a 30-min period in absence of agonist display a fully-recovered cyclic AMP response to isoproterenol. Details of the desensitization/resensitization protocols are described elsewhere [19].

## Abbreviations

AKAP: A-kinase anchoring protein; AKAP5: AKAP79; AKAP12: gravin, AKAP250, SSECKS; A-kinas, PKA: protein kinase A; Erk: mitogen-activated protein kinase, extracellular signal-regulated kinase; GPCR: G protein-coupled receptor; SDS-PAGE: sodium dodecyl sulfate-polyacrylamide gel electrophoresis.

## Competing interests

The authors declare that they have no competing interests.

## Authors' contributions

JT designed the study, performed the experiments, gathered the data, and outlined the manuscript. CCM participated in the evolution of the study design, the interrogation of the experimental results, and helped to draft the manuscript. Both authors read and approved the final manuscript.
